# Recognizing Recurrence: History Over Symptoms in Metastatic Melanoma

**DOI:** 10.7759/cureus.80532

**Published:** 2025-03-13

**Authors:** John Solomon, Niyousha Naderi, Qui Cheun Ng, Eric Jacobson

**Affiliations:** 1 Radiology, KPC Health, Hemet, USA; 2 Diagnostic Radiology, KPC Health, Hemet, USA

**Keywords:** brain metastasis, melanoma recurrence, metastatic melanoma, surgical resection, t1 hyperintense, vasogenic edema

## Abstract

Imaging findings of diseases are not always very typical. A comprehensive history helps the radiologist make a differential diagnosis list based on likelihood and provides the most helpful report. In this paper, we presented the case of a 41-year-old male with a history of stage IV melanoma with prior brain metastasis and resection who came to the emergency department reporting intermittent headaches, dizziness, vomiting, and a loss of appetite. Diagnostic imaging revealed a right frontal lobe mass concerning recurrent melanoma metastasis. Neurosurgery was consulted, and it was decided to resect the mass. Immunohistochemical staining of the tumor cells confirmed that the lesion was of melanocytic origin. Postoperatively, the patient showed significant improvement in symptoms, with no immediate complications, and continued adjuvant therapies based on the surgical and pathological findings. This case stresses the importance of remaining vigilant for new or worsening symptoms in patients with a history of melanoma, given the aggressive nature of this cancer and the high recurrence rate.

## Introduction

Melanoma commonly presents clinically in the fourth or fifth decade of life. It is characterized by asymmetrical pigmented skin lesions with irregular borders, varying colors, and a diameter greater than 6 mm. It is a highly aggressive cancer from pigment-producing melanocytes found in the skin, eyes, inner ear, leptomeninges, heart, and mucosa [1]. Embryonic neural crest cells give rise to melanocytes, sympathetic neuroblasts, and chromaffin cells. The extensive migration of these cells increases the susceptibility of their derivatives to aggressive carcinogenesis, leading to their respective cancer types: melanomas, neuroblastomas, and malignant pheochromocytomas.

The shared embryonic origin and receptor profiles between melanocytes and other neural cells may partially explain why melanoma has a propensity to metastasize to the brain. Melanoma cells exploit similar pathways and receptors that neural cells use to invade and thrive in the brain microenvironment. Adhesion molecules such as MUC1, VCAM1, and VLA-4 facilitate their adhesion to the brain endothelium. Consequently, neurotrophins (NT-3) and their receptors (TrkC) promote invasion through the production of the extracellular matrix-degrading enzyme heparinase [2].

Although melanoma accounts for approximately 1% of all skin cancers, it is the most responsible for skin cancer mortality due to its high metastatic potential [3]. This case highlights the importance of vigilance for new symptoms indicating recurrence, which affects patient outcomes.

## Case presentation

A 41-year-old male with a known history of stage IV melanoma and prior brain metastasis resection with chemoradiation, currently undergoing immunotherapy, presented to the emergency department with intermittent headaches, dizziness, vomiting, and loss of appetite for one week. He had also undergone a resection of a posterior neck lesion, assumed to have been the primary site of melanoma.

Examination revealed a left posterior neck scar and a nodule palpated on the left side of the neck. Bradycardia without murmurs was noted on the cardiovascular examination. Neurologically, the patient was alert and oriented with no focal deficits. There was no evident lymphadenopathy. All other examination findings were normal.

**Figure 1:**
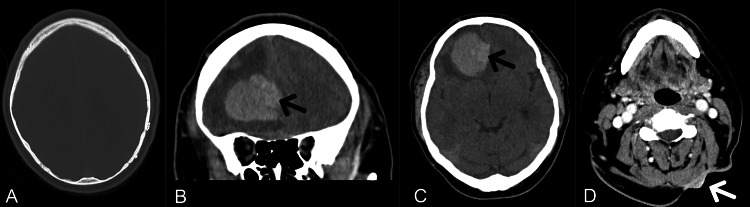
(A-C) CT of the head without contrast: The axial bone window (A) shows the previous left parietal craniotomy. The coronal view (B) and axial view (C) reveal an ovoid-shaped, heterogeneous, hyperdense mass in the right frontal lobe, along with an area of slightly greater hyperdensity medially within the mass, suspicious for hemorrhage (black arrows). There is also edema surrounding the mass, with mass effect on the frontal horns and displacement of the falx (midline shift) to the left. (D) CT of the neck with contrast: A soft tissue mass is seen in the posterior aspect of the neck (white arrow), accompanied by skin thickening.

**Figure 2:**
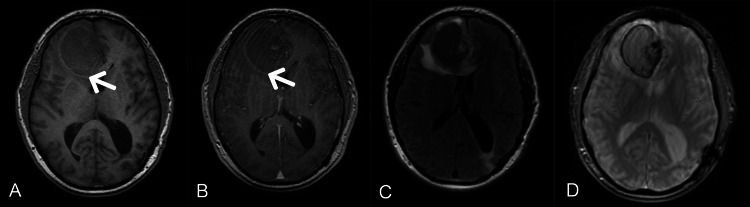
MRI brain: The T1-weighted axial image without contrast (A) shows peripheral T1 hyperintensity and heterogeneous mildly increased T1 signal internally. T1 post-contrast (B) reveals an ovoid right frontal lobe mass, predominantly isointense to the cortex, with peripheral T1 hyperintensity (white arrows) and mild central nodular heterogeneous enhancement. The T2 FLAIR (C) demonstrates the mass, which mostly exhibits low signal, with a nodule of increased signal. There is associated peri-lesional vasogenic edema and mass effect, with midline shift involving the bilateral frontal horns of the lateral ventricles. The gradient recalled echo (GRE) image (D) shows peripheral and internal foci of blooming, suggestive of hemorrhage.

**Figure 3:**
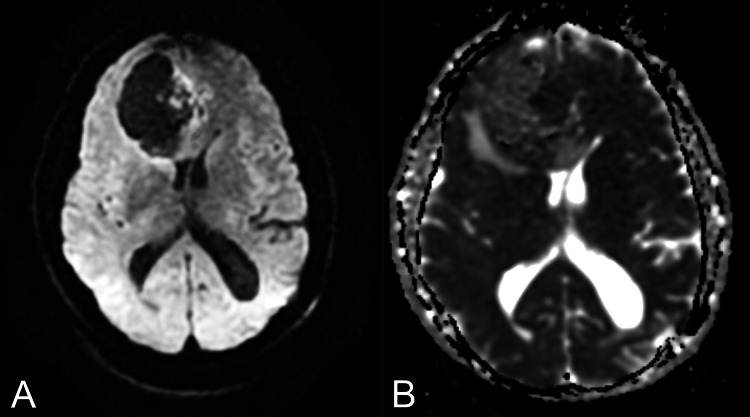
The axial diffusion-weighted image (A) and axial apparent diffusion coefficient (ADC) image (B) demonstrate a focus of heterogeneous restricted diffusion medially.

Laboratory results showed a mild elevation in white blood cell (WBC) count with neutrophil dominance, indicating an active immune response to the cancer. Other lab results were non-contributory to the case. A CT scan of the brain (Figure 1) with contrast revealed a large frontal lobe intracranial mass measuring approximately 51 x 38 mm, accompanied by significant edema and a mild midline shift. A soft tissue mass in the posterior left neck measured approximately 23 x 8 mm, with continuous skin thickening but no definite invasion of adjacent musculature. MRI was obtained for further characterization of the brain lesion (Figure 2 and Figure 3).

The patient was evaluated and found eligible for surgical resection of the lesion. The decision was based on the location and accessibility of the mass, the patient’s overall health status, and the potential for improved neurological outcomes post-surgery. The surgical approach aimed to remove as much of the tumor as safely possible to alleviate symptoms, reduce mass effect, and improve quality of life. The mass stained positive for HMB-45, S100, and SOX10, and negative for CK7, CK20, GFAP, PR, and SSTR2 with a Ki-67 proliferation rate of 40-50%. The diagnosis of metastatic melanoma with hemorrhage/hematoma was confirmed based on these findings. Postoperatively, the patient was closely monitored for complications and instructed to follow-up with his oncologist.

## Discussion

Malignant melanoma is known for its high potential for metastasis, and this case underscores several critical points in managing advanced melanoma. This patient’s initial presentation could have been misdiagnosed as an atypical migraine or even a tension headache. The history, however, of metastatic melanoma should prompt clinicians to obtain proper imaging.

The specific imaging characteristics of intracranial melanoma metastatic lesions are often due to the presence of melanin, intra-lesional hemorrhage, and vascular proliferation. In MR imaging, intracranial malignant melanomas typically appear with high signal intensity on the T1-weighted images and low signal intensity on the T2-weighted images [4]. However, the presented case demonstrated a somewhat atypical appearance as the mass showed less than expected T1 sequence intensity. A rare case of amelanotic cystic presentation of intracranial metastatic melanoma was previously reported by Ogawa et al. in a 51-year-old female with an occipital lesion, which showed low intensity on T1-weighted images and high intensity on T2-weighted images [5]. The role of MR imaging is pivotal in characterization of the melanoma metastasis and its correlation with the patient's history makes the noninvasive diagnosis possible.

In the case presented here, diagnostic imaging confirmed a right frontal lobe mass, which could explain the patient’s symptoms. The observed bradycardia may have been a vasopressor response (Cushing reflex). The pathology report confirmed melanoma origin and a high-grade cellular proliferation rate. The laboratory finding of a high neutrophil-to-lymphocyte ratio can also be an independent prognostic indicator of poor survival, as the literature suggests [6].

Postoperatively, the patient showed significant symptom improvement with no immediate complications. Studies have shown that the recurrence rate of metastatic melanoma following resection can range from approximately 17% to 54.6%, with a median time to recurrence around 4.6 months to 2 years [7,8,9].

## Conclusions

Clinicians must be vigilant for signs of recurrence in patients with a history of metastasis, regardless of previous treatment, especially when presenting to the emergency department with new or worsening symptoms, due to the importance of early detection and treatment. Additionally, a comprehensive and relevant history helps the radiologist provide a differential diagnosis based on likelihood, particularly when encountering somewhat atypical presentations of the disease, in order to provide the best care possible for the patient.

## References

[1] Cichorek M, Wachulska M, Stasiewicz A, Tymińska A (2013). Skin melanocytes: biology and development. Postepy Dermatol Alergol.

[2] Denkins Y, Reiland J, Roy M (2004). Brain metastases in melanoma: roles of neurotrophins. Neuro Oncol.

[3] Yaar M, Park HY (2012). Melanocytes: a window into the nervous system. J Invest Dermatol.

[4] Isiklar I, Leeds NE, Fuller GN, Kumar AJ (1995). Intracranial metastatic melanoma: correlation between MR imaging characteristics and melanin content. AJR Am J Roentgenol.

[5] Ogawa R, Aoki R, Hyakusoku H (2003). A rare case of intracranial metastatic amelanotic melanoma with cyst. J Clin Pathol.

[6] Zaragoza J, Caille A, Beneton N, Bens G, Christiann F, Maillard H, Machet L (2016). High neutrophil to lymphocyte ratio measured before starting ipilimumab treatment is associated with reduced overall survival in patients with melanoma. Br J Dermatol.

[7] Owen CN, Shoushtari AN, Chauhan D (2020). Management of early melanoma recurrence despite adjuvant anti-PD-1 antibody therapy(☆). Ann Oncol.

[8] von Schuckmann LA, Hughes MC, Ghiasvand R (2019). Risk of melanoma recurrence after diagnosis of a high-risk primary tumor. JAMA Dermatol.

[9] Liang C, Hu W, Li J, Zhang X, Zhou Z, Liang Y (2021). Early time to recurrence predicts worse survival in patients with localized or regionally advanced cutaneous melanoma. Dermatol Ther.

